# Supplementary material for: Co-expression of CD39 and CD103 identifies tumor-reactive CD8 T cells in human solid tumors
Source: Nat Commun. 2018 Jul 13;9:2724. doi: 10.1038/s41467-018-05072-0 (PMC6045647; doi:10.1038/s41467-018-05072-0)
Supplement: Supplementary file 1 — Supplementary Information [file 41467_2018_5072_MOESM1_ESM.pdf]

Co-expression of CD39 and CD103 identifies tumor-reactive CD8 T cells in human solid tumors

Duhen et al.

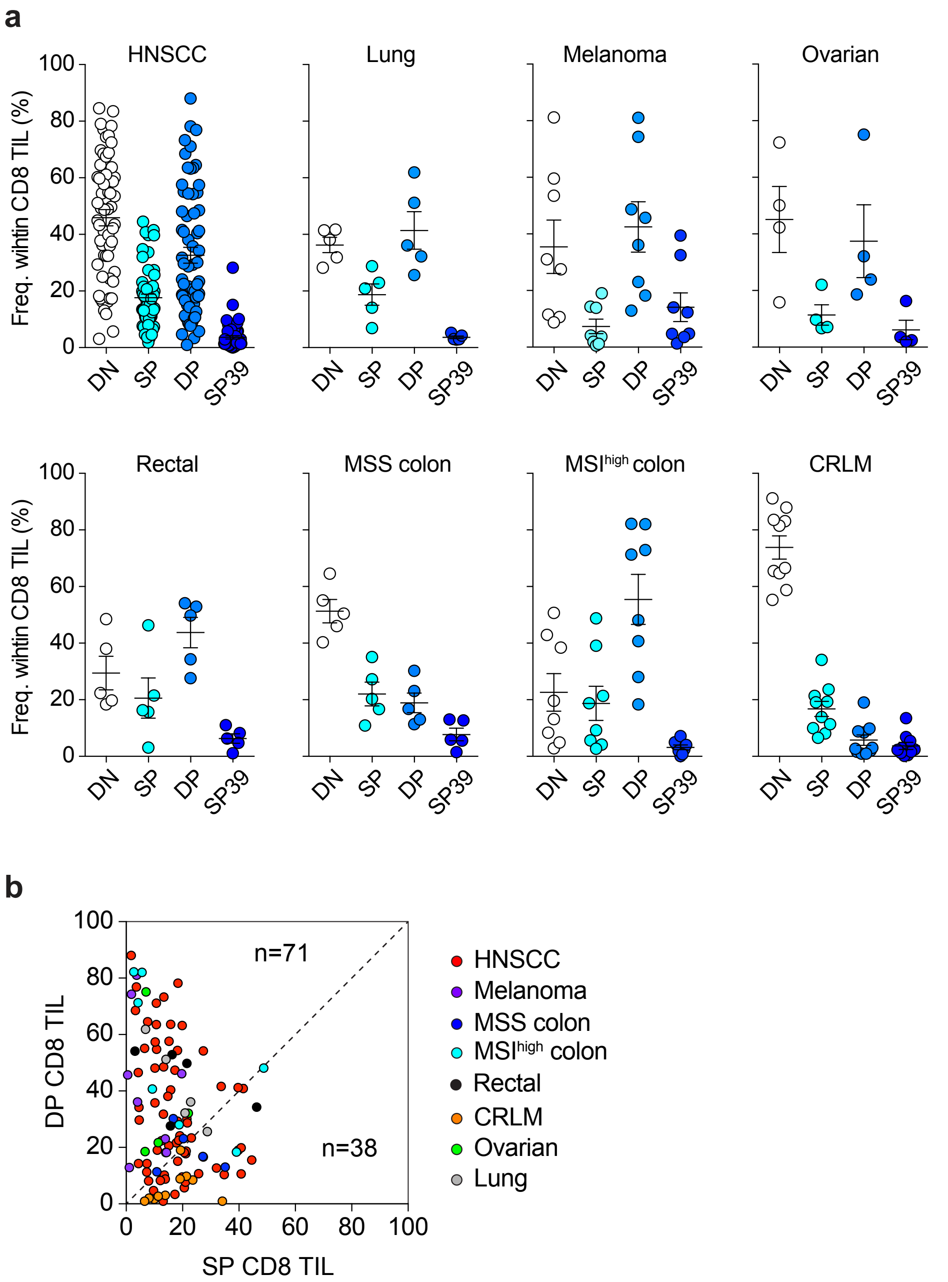

Supplementary Figure 1. Frequencies of CD8 TIL populations identified by the expression of CD103 and CD39 in different human cancer histologies. (**a**) Ex vivo flow cytometric analysis of the expression of CD103 and CD39 on CD8 TILs isolated from patients with HNSCC (n=65), lung cancer (n=5), melanoma (n=8), ovarian cancer (n=4), rectal cancer (n=5), colon cancer (MSS and MSI^{high}, n=5 and n=8 respectively) and CRLM (n=10). Combinations of expression of CD103 and CD39 are indicated (DN, SP, DP and SP39). (**b**) Comparison of the frequency of SP CD8 and DP CD8 TILs within each patient across different solid tumors. Each symbol represents one patient and each tumor type is highlighted with a unique color. Small horizontal lines indicate mean ±SEM.

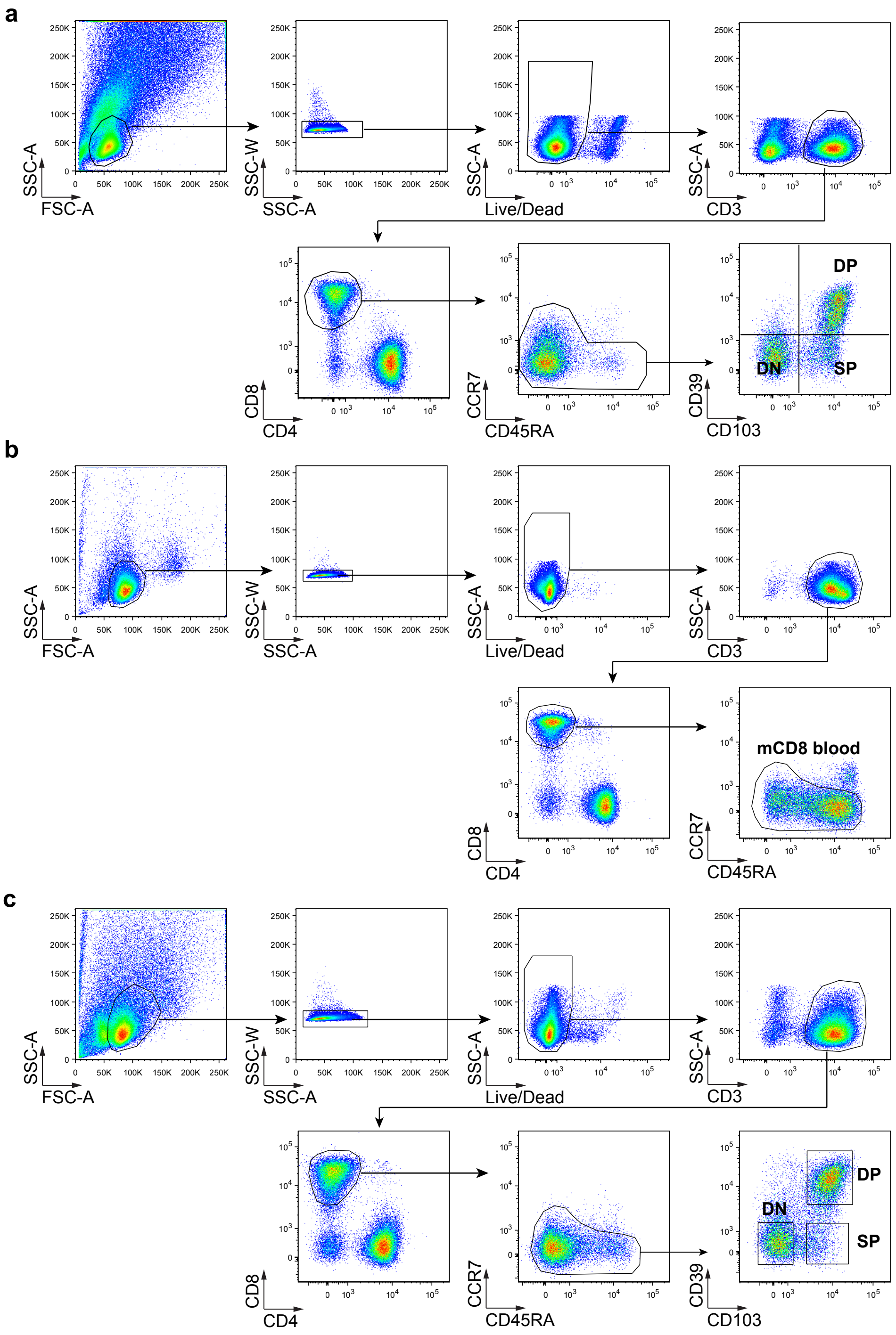

Supplementary Figure 2. Gating strategies (**a**) Gating strategy to analyze the phenotype of CD8 TILs (Fig. 1c-f and Fig. 3a-e). (**b**) Gating strategy to sort memory CD8 T cells from peripheral blood (mCD8 blood) or normal LN (mCD8 LN) for TCR repertoire analysis (Fig. 5a and c). (**c**) Gating strategy to sort DN, SP and DP CD8 T cells from primary tumor or metastatic LN for gene-expression analysis (Fig. 2a-e), TCR repertoire analysis (Fig. 5a-d) and tumor reactivity experiments (Fig. 6a-e).

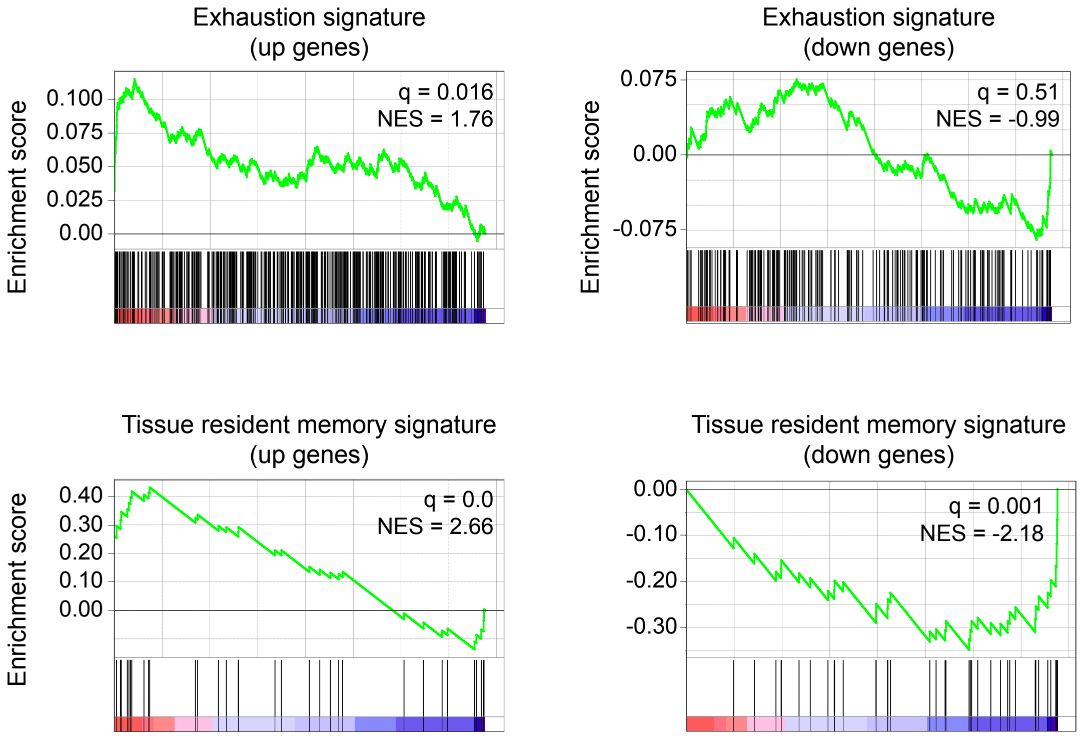

Supplementary Figure 3. Comparison of the gene expression profile of DP CD8 TILs versus SP CD8 TILs. Gene Set Enrichment Analysis (GSEA) of the "CD8 T cell exhaustion" and "tissue resident memory T cell" gene sets in the trancriptome of DP CD8 TILs versus that of SP CD8 TILs presented as the normalized enrichment score (NES) for the gene set as the analysis "walks down" the ranked list of genes (reflective of the degree to which the gene set is over-represented at the top or bottom of the ranked list of genes) and the position of the gene set members (black vertical lines).

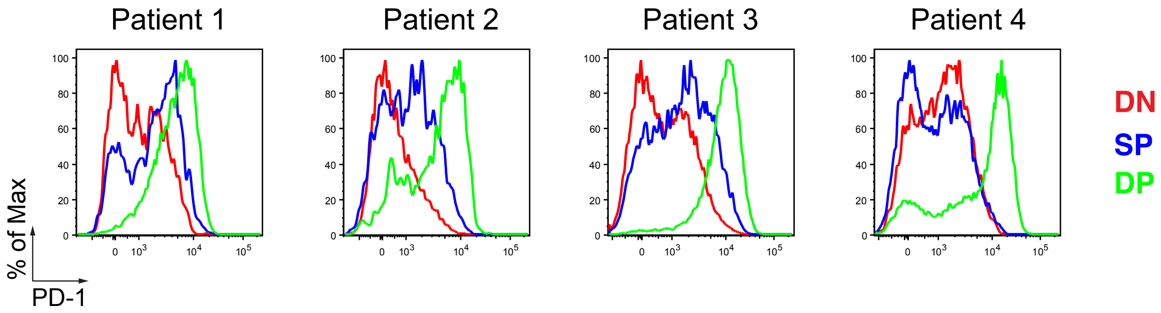

Supplementary Figure 4. PD-1 expression on DN, SP and DP CD8 TILs. Ex vivo flow cytometric analysis of the expression of PD-1 on CD8 TILs isolated from 4 different HNSCC patients (colors match key).

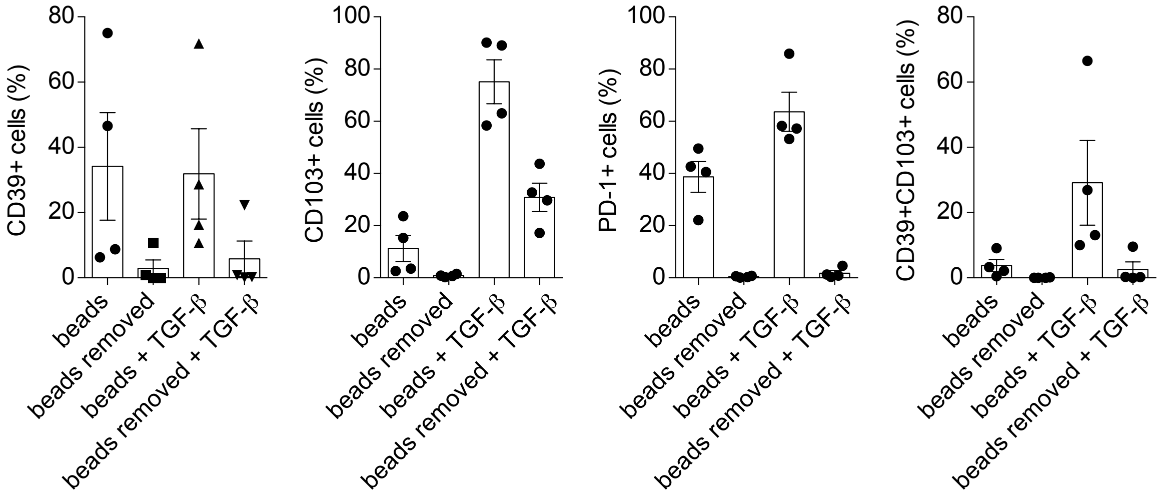

Supplementary Figure 5. Expression of CD39 and CD103 on CD8 T cells requires sustained stimulation in TGF-β-rich milieu. CD39, CD103 and PD-1 expression on sorted naïve CD8 T cells from peripheral blood (n = 4). Cells were stimulated with CD3/CD28 coated beads in the presence or absence of TGF-β (2 ng/ml) and expression of CD39, CD103 and PD-1 was analyzed by flow cytometry at day 9. When indicated the beads were removed after 24h of culture. Small horizontal lines indicate mean ±SEM.

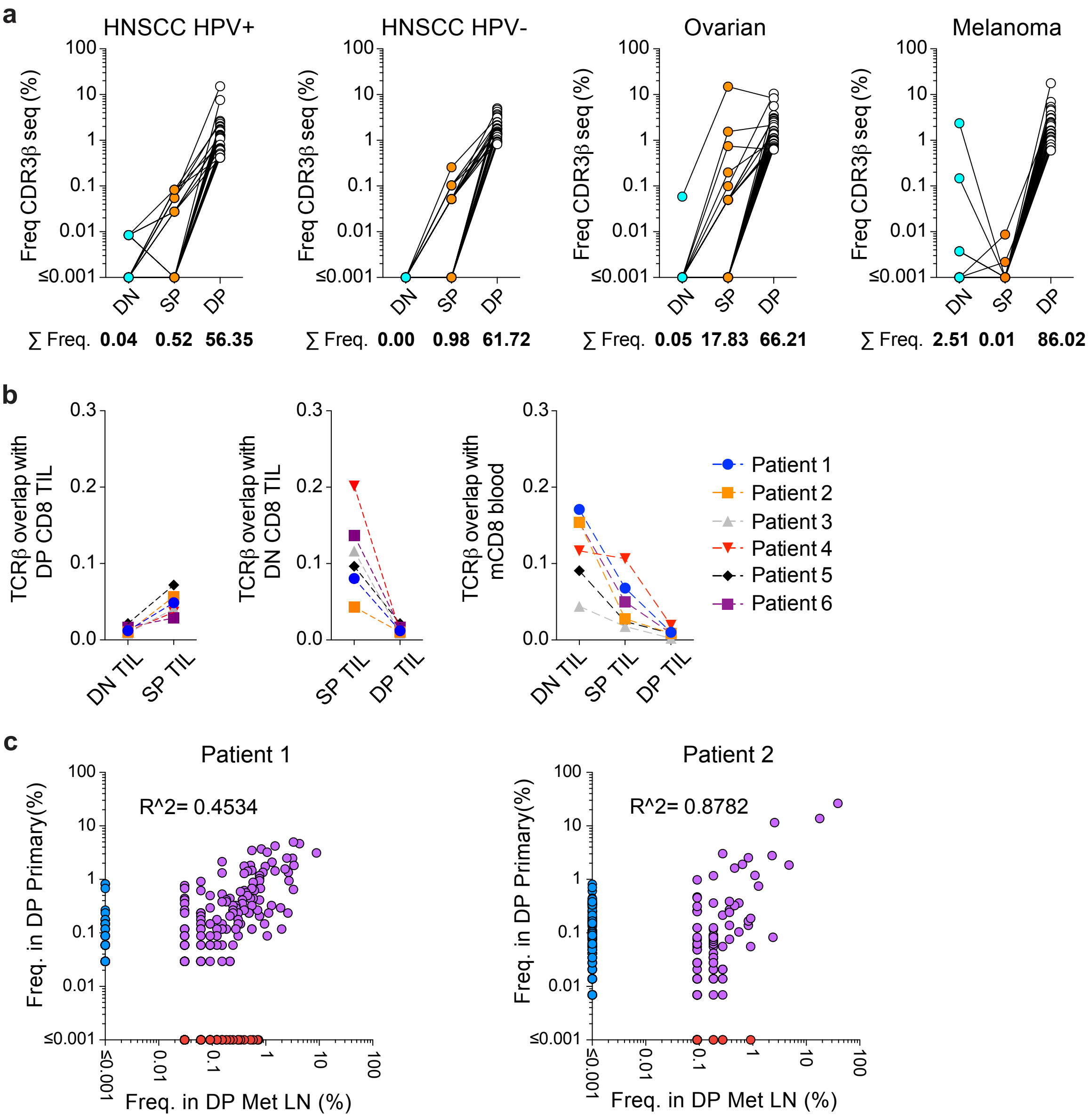

Supplementary Figure 6. DP CD8 TILs display a distinct TCRβ repertoire.
(**a**) Frequency of the 30 most frequent clonotypes in the DP CD8 TILs in a HPV+ HNSCC, a HPV- HNSCC, an ovarian tumor and a melanoma tumor. Their frequency in the DN, SP and DP CD8 TILs is represented. Each dot represents 1 unique TCRβ clonotype. Cumulative frequencies (Σ Freq.) of the clonotypes in each of the populations are shown below. (**b**) Similarity between the TCR repertoires of CD8 T cell subsets was measured using the Jaccard index on 6 cancer patients. (**c**) The 500 most frequent CD8 TIL clonotypes are plotted based on their frequency in DP CD8 TILs in the primary tumor and in a metastatic LN in 2 HNSCC patients. Each dot represents 1 unique TCRβ clonotype. Dots on the axis indicate the clonotypes detected within a single repertoire; purple dots indicate clonotypes shared between the two CD8 T cell populations. The Pearson correlation coefficient (R^2) was calculated by GraphPad Prism 6.

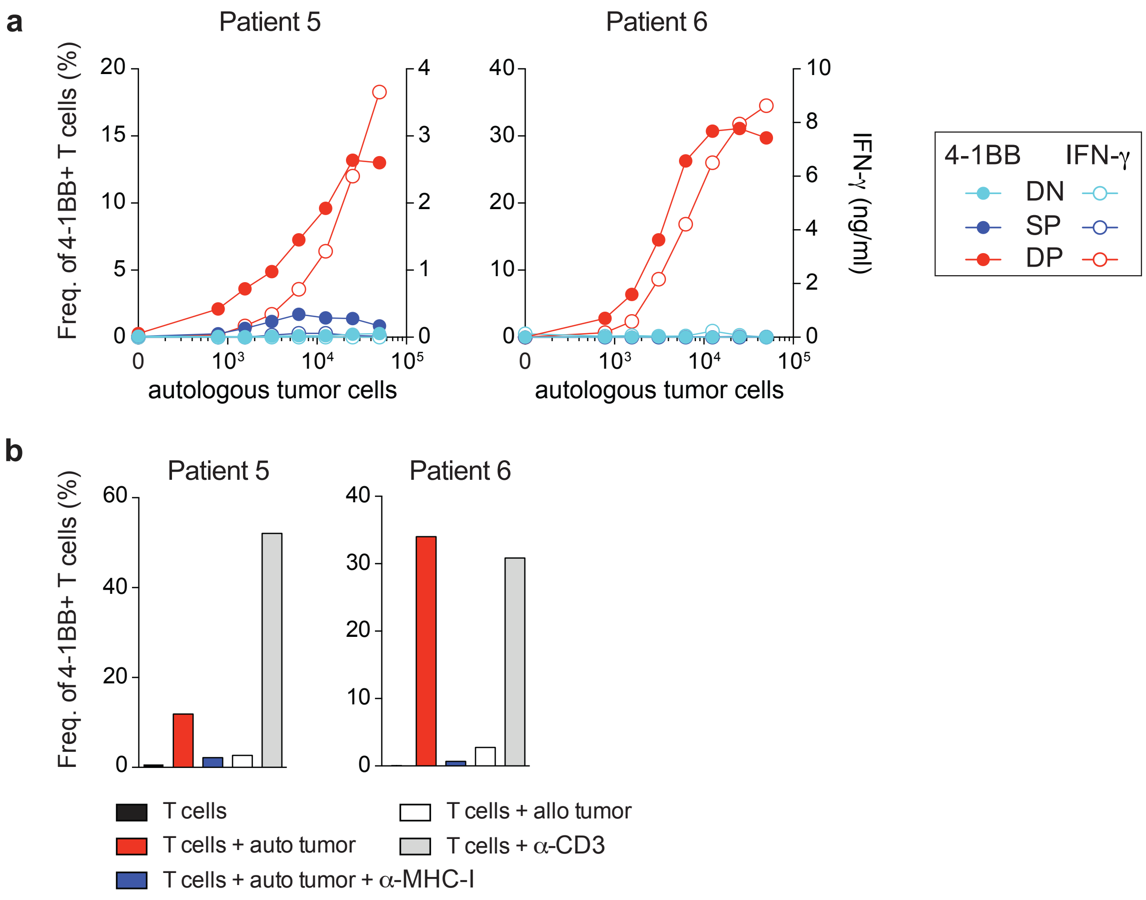

Supplementary Figure 7. 4-1BB expression and IFN-γ secretion by expanded CD8 T cell subsets in response to tumor cells. DN, SP and DP CD8 TILs were sorted from tumor digest and expanded in vitro. (**a**) Expanded CD8 TILs were tested for tumor reactivity by cultivating them for 20h with increasing numbers of autologous tumor cells, and tumor recognition was assessed by measuring the frequency of 4-1BB expression (filled symbols) and IFN-γ secretion (open symbols). Results are shown for two HNSCC patients. (**b**) Reactivity of DP CD8 TILs was confirmed by culture with autologous tumor cells with and without MHC-I blocking antibody, allogeneic tumor cells, and plate-bound anti-CD3. The up-regulation of 4-1BB after 20h is shown for two HNSCC patients.

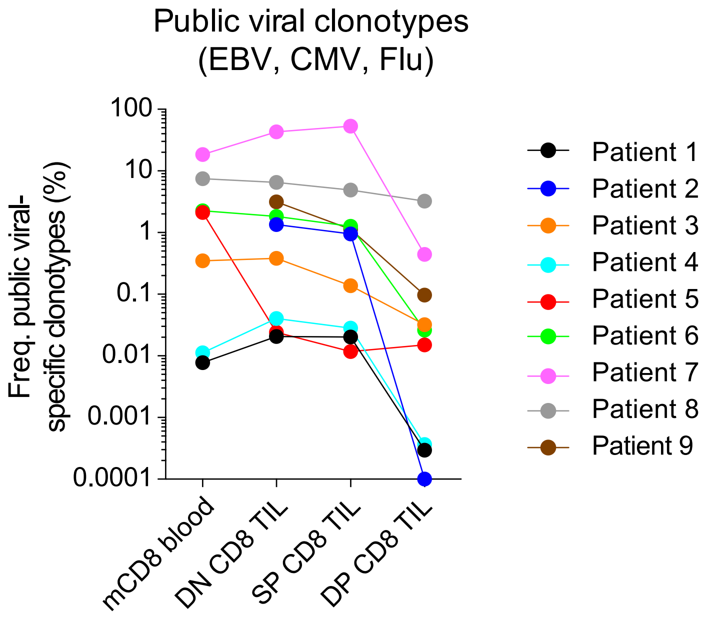

Supplementary Figure 8. Frequency of public viral-specific clonotypes in CD8 TIL subsets. The TCRβ repertoires of memory CD8 T cells isolated from the blood (mCD8 blood) and DN, SP and DP CD8 T cells isolated from the tumor were queried against public TCR sequences specific for EBV, CMV and Flu virus hosted by the VDJ database (Shugay M et al. VDJdb: a curated database of T-cell receptor sequences with known antigen specificity. Nucleic Acids Research 2017, gkx760). The combined frequency of TCR clonotypes specific for those viruses present in the indicated cell type is represented in the graph. Nine patients were analyzed and each patient is represented by a unique color. For patient 2 and 9 no blood sample was obtained.

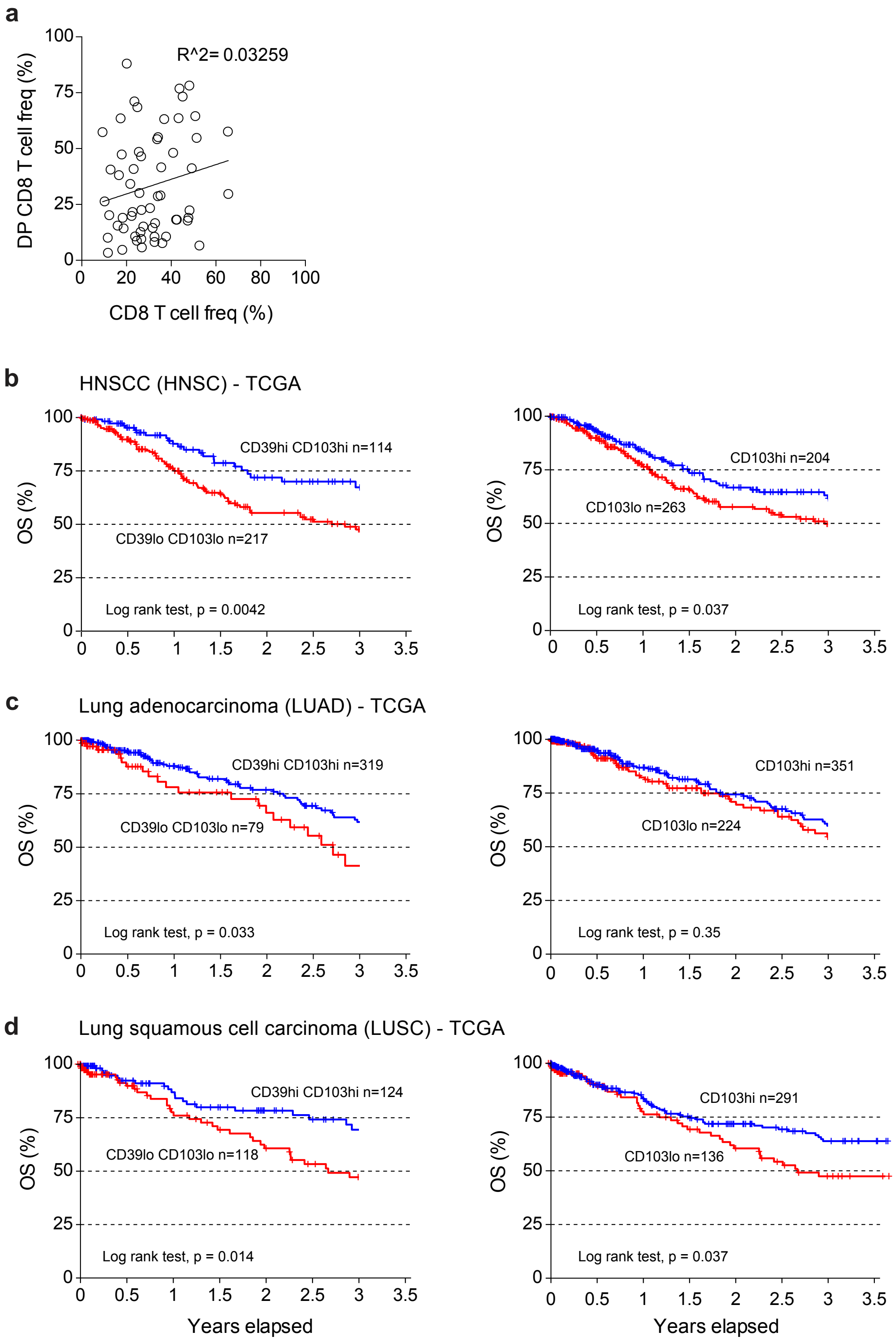

Supplementary Figure 9. Frequency of DP CD8 TILs and cancer patient survival. (**a**) Correlation between the frequency of CD8 T cells among total T cells and the frequency of DP CD8 cells among total CD8 TILs (HNSCC, n=59). (**b**) Survival of patients with HNSCC (HNSC) from the TCGA dataset, classified on the basis of expression of *ENTPD1* and *ITGAE* transcripts into CD39hi CD103hi tumors or CD39lo

CD103lo tumors or CD103hi tumors and CD103lo tumors. (**c**) Similar analysis performed on patients with lung adenocarcinoma (LUAD) from the TCGA dataset. (**d**) Similar analysis performed on patients with lung squamous cell carcinoma (LUSC) from the TCGA dataset. The Pearson correlation coefficient (R^2) was calculated by GraphPad Prism 6 (**a**). The log-rank test was used to compare survival curves and *p*-values less than 0.05 were considered significant (**b**, **c** and **d**).

Supplementary Table 1. List of the top 100 genes that contribute the most to PC1 of the PCA plot. The weight of each gene is indicated in the table below.

Gene	Weight	Gene	Weight
POU2AF1	3.989	STMN1	2.773
CXCL13	3.925	SLC43A3	2.686
LOC100509764	3.883	SMC2	2.681
MYO7A	3.842	POLQ	2.645
C11orf75	3.780	BIRC5	2.625
HIST1H1B	3.756	GALNT2	2.613
CTLA4	3.750	VDR	2.613
NCRNA00158	3.747	CHAF1A	2.605
TNFRSF9	3.726	PAQR4	2.601
ENTPD1	3.724	FAM3C	2.549
GZMB	3.714	TNFSF4	2.505
NHS	3.714	CCDC50	2.474
ITGAE	3.676	UHRF1	2.461
HMOX1	3.650	BCL2L11	2.459
KRT81	3.630	LMCD1	2.435
TYMS	3.622	C12orf48	2.368
CHST12	3.618	TCF7	-2.333
PHEX	3.578	PLAC8	-2.344
MKI67	3.558	PZP	-2.351
HAVCR2	3.557	FAM65B	-2.354
HIST2H2AB	3.550	SPOCK2	-2.357
ASPM	3.504	SLC44A1	-2.366
LAYN	3.496	C19orf22	-2.379
ADCY3	3.491	PIK3R1	-2.388
KIF23	3.482	MYBL1	-2.398
ASF1B	3.463	P2RY8	-2.454
AKAP5	3.458	BEX5	-2.456
MGC29506	3.444	YPEL5	-2.462
HIST1H3G	3.439	BACH2	-2.462
HIST1H4L	3.435	ZNF204P	-2.518
KIR2DL4	3.429	MGAT4A	-2.524
NR5A2	3.416	IL7R	-2.540
NAB1	3.415	CCR4	-2.557
HIST1H2AI	3.380	A2M	-2.570
SIRPG	3.339	S1PR5	-2.573
UBE2C	3.335	LGR6	-2.597
LOC652797	3.320	EPHA4	-2.611
RBPJ	3.289	PDCD4	-2.624
RAD51AP1	3.279	LDLRAP1	-2.659
CRIM1	3.262	SLC26A11	-2.659
AFAP1L2	3.250	S1PR1	-2.662
FAM111B	3.205	KLF3	-2.663
GPR56	3.190	PTPRM	-2.672
HECTD2	3.146	IFNGR1	-2.682
CD200	3.102	KLF2	-2.701
PTPRN2	3.079	SORL1	-2.707
NEIL3	3.075	LSR	-2.715
ETV1	3.022	MYO1D	-2.715
CHEK1	2.914	CCR7	-2.732
FABP5	2.809	SELL	-2.755

Supplementary Table 2. Patient characteristics for HNSCC patients (n = 62). Fisher exact tests, and F or nonparametric Kruskal-Wallis Rank Sum test were performed for comparing DP low and DP high (cutoff by mean = 32.9%) by categorical, and continuous variables, respectively.

	**Total (N = 62)**	**DP Low (N = 36)**	**DP High (N = 26)**	
**Variables**	**No (%)**	**No (%)**	**No (%)**	***p*-value**
**Age, years**				
Median (Q1, Q3)	62.0 (54.3, 69.8)	60.5 (53.8, 68.0)	65.5 (59.3, 70.8)	0.17
Mean (SD)	62.5 (12.3)	61.1 (11.3)	64.3 (13.6)	0.32
**Gender**				0.23
Female	15 (24)	11 (31)	4 (15)	
Male	47 (76)	25 (69)	22 (85)	
**HPV Status**				0.21
Negative	30 (48)	20 (56)	10 (38)	
Positive	32 (52)	16 (44)	16 (62)	
**Primary Site**				0.53
Hypopharynx	3 (5)	2 (6)	1 (4)	
Larynx	5 (8)	4 (11)	1 (4)	
Oral	20 (32)	13 (36)	7 (27)	
Oropharynx	34 (55)	17 (47)	17 (65)	
**Smoking**				0.43
Never	23 (37)	15 (42)	8 (31)	
Ever	39 (63)	21 (58)	18 (69)	
**Stage**				0.02
I-III	16 (26)	5 (14)	11 (42)	
IV (IVa, IVb or IVc)	46 (74)	31 (86)	15 (58)	

Supplementary Table 3. Univariate and multivariable OS analysis using Cox-proportional model with Firth's penalized likelihood (n = 62).

	Univariate Analysis				Multivariable Analysis			
**Variables**	**HR**	**95% Lower CI**	**95% Lower CI**	***p*-value**	**HR**	**95% Lower CI**	**95% Lower CI**	***p*-value**
**DP CD8 (%), cutoff by mean (32.9%)**								
Low								
High	0.24	0.05	0.80	0.02	0.23	0.04	0.96	0.04
**HPV Status**								
Negative								
Positive	0.15	0.03	0.49	0.001	0.12	0.02	0.43	0.001
**Age at Surgery**	1.00	0.96	1.04	0.98	0.98	0.94	1.02	0.37
**Gender**								
Female								
Male	0.63	0.23	1.93	0.40	1.48	0.49	4.89	0.49
**Smoking Status**								
Never								
Ever	1.47	0.53	4.93	0.48	1.37	0.46	4.78	0.58
**Stage**								
I-III								
IV (IVa, IVb or IVc)	2.03	0.62	10.34	0.27	1.40	0.34	8.37	0.66

Note: Taking DP CD8 as a continuous variable with log(2) transformed (based on all 62 HNSCC patients):

Taking DP CD8 as a continuous variable with log (2) transformed [i.e., log(DP CD8, 2)], the univariate analysis shows that there is no significant association of DP CD8 with overall survival [HR (95% CI) = 0.71 (0.47, 1.07), *p*-value = 0.10]. However, Multivariable analysis shows that after controlling for HPV status, age, gender, smoking status and stage, the association of DP CD8 with overall survival [HR (95% CI) = 0.63 (0.38, 1.03)] is almost significant (*p*-value = 0.07).

Supplementary Table 4. Univariate and multivariable OS analysis for HPV-negative patients using Cox-proportional model with Firth's penalized likelihood (n = 30).

	**Univariate Analysis**				**Multivariable Analysis**			
**Variables**	**HR**	**95% Lower CI**	**95% Lower CI**	***p*-value**	**HR**	**95% Lower CI**	**95% Lower CI**	***p*-value**
**DP CD8 (%), cutoff by mean (32.9%)**								
Low								
High	0.16	0.02	0.68	0.01	0.07	0.003	0.54	0.01
**Age at Surgery**	0.98	0.95	1.02	0.30	0.96	0.90	1.00	0.07
**Gender**								
Female								
Male	1.02	0.35	3.21	0.97	1.60	0.50	5.64	0.43
**Smoking Status**								
Never								
Ever	2.13	0.63	10.95	0.24	3.06	0.78	18.88	0.12
**Stage**								
I-III								
IV (IVa, IVb or IVc)	2.08	0.61	10.74	0.26	0.93	0.19	6.42	0.93

Note: Taking DP CD8 as a continuous variable with log(2) transformed (based on all 30 HNSCC HPV-negative patients):

Taking DP CD8 as a continuous variable with log(2) transformed [i.e., log(DP CD8, 2)], the univariate analysis shows that the hazard of death significantly decreased 48% with 2-fold increase of DP CD8[HR (95% CI) = 0.52 (0.29, 0.89), *p*-value = 0.02]. Multivariable analysis shows that after controlling for HPV status, age, gender, smoking status and stage, the hazard of death significantly decreased 45% with 2-fold increase of DP CD8 [HR (95% CI) = 0.55 (0.32, 0.95), *p*-value = 0.03.

Supplementary Table 5. Estimate OS at 1- year, 2-year and 3-year with 95% confidence interval (CI) by DP Low versus High.

DP CD8	# Events/Total	Estimate OS (95% CI) (%)			*p*-value*
		1-year	2-year	3-year	
**DP Low**	13 / 36	74 (61, 90)	56 (40, 80)	56 (40, 80)	0.02
**DP High**	2 / 26	96 (88, 100)	89 (75, 100)	89 (75, 100)	

*log-rank test

Supplementary Table 6. Estimate OS at 1- year, 2-year and 3-year with 95% confidence interval (CI) by DP Low versus High for HPV-negative patients.

DP CD8	# Events/Total	Estimate OS (95% CI) (%)			*p*-value*
		1-year	2-year	3-year	
**DP Low**	12 / 20	58 (40, 85)	26 (10, 71)	26 (10, 71)	0.01
**DP High**	1 / 10	100 (100, 100)	86 (63, 100)	86 (63, 100)	

*log-rank test
